# Poly[tris­(dimethyl­formamide)(μ_3_-2,4,6-triiodobenzene-1,3,5-tricarboxyl­ato)samarium(III)]

**DOI:** 10.1107/S1600536813003358

**Published:** 2013-02-13

**Authors:** Bin Yan, Daopeng Sheng, Yanzhao Yang

**Affiliations:** a Key Laboratory for Special Functional Aggregated Materials of the Education Ministry, School of Chemistry and Chemical Engineering, Shandong University, Jinan, Shandong 250100, People’s Republic of China

## Abstract

In the title compound, [Sm(C_9_I_3_O_6_)(C_3_H_7_NO)_3_]_*n*_, the Sm^III^ atom is coordinated by nine O atoms, *viz.* six carboxyl­ate O atoms from three 2,4,6-triiodobenzene-1,3,5-tricarboxyl­ate (I_3_BTC) ligands and three O atoms from three *N*,*N*-dimethyl­formamide (DMF) mol­ecules. Each I_3_BTC ligand bridges three Sm^III^ atoms, generating a three-dimensional metal-organic framework structure. The asymmetric unit contains one Sm^III^ ion and one I_3_BTC anion, both situated on a threefold axis, and one DMF mol­ecule in a general position.

## Related literature

For applications of compounds with metal-organic framework structures (MOFs), see: Nakanishi & Tanaka (2007 ▶); Phan *et al.* (2010 ▶); Suib *et al.* (2008 ▶). For related structures, see: Daiguebonne *et al.* (2002 ▶); Han *et al.* (2012 ▶); Lu *et al.* (2008 ▶); Serre *et al.* (2004 ▶).
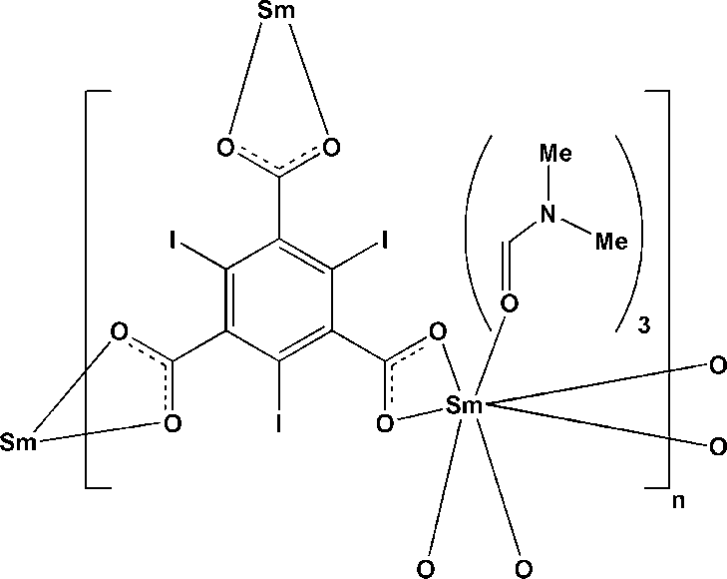


## Experimental

### 

#### Crystal data

[Sm(C_9_I_3_O_6_)(C_3_H_7_NO)_3_]
*M* *_r_* = 954.43
Cubic, 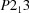

*a* = 14.1341 (16) Å
*V* = 2823.6 (6) Å^3^
*Z* = 4
Mo *K*α radiation
μ = 5.41 mm^−1^
*T* = 295 K
0.16 × 0.15 × 0.15 mm

#### Data collection

Bruker APEXII CCD area-detector diffractometer
Absorption correction: multi-scan (*SADABS*; Bruker, 2005 ▶) *T*_min_ = 0.478, *T*_max_ = 0.498
5119 measured reflections
2143 independent reflections
2026 reflections with *I* > 2σ(*I*)
*R*_int_ = 0.020

#### Refinement

*R*[*F* ^2^ > 2σ(*F* ^2^)] = 0.021
*wR*(*F* ^2^) = 0.049
*S* = 1.03
2143 reflections
106 parameters
H-atom parameters constrained
Δρ_max_ = 0.72 e Å^−3^
Δρ_min_ = −0.62 e Å^−3^
Absolute structure: Flack (1983 ▶), 943 Friedel pairs
Flack parameter: 0.02 (2)

### 

Data collection: *APEX2* (Bruker, 2005 ▶); cell refinement: *SAINT* (Bruker, 2005 ▶); data reduction: *SAINT*; program(s) used to solve structure: *SIR97* (Altomare *et al.*, 1999 ▶); program(s) used to refine structure: *SHELXL97* (Sheldrick, 2008 ▶); molecular graphics: *SHELXTL* (Sheldrick, 2008 ▶); software used to prepare material for publication: *WinGX* (Farrugia, 2012 ▶).

## Supplementary Material

Crystal structure: contains datablock(s) I, global. DOI: 10.1107/S1600536813003358/cv5378sup1.cif

Structure factors: contains datablock(s) I. DOI: 10.1107/S1600536813003358/cv5378Isup2.hkl

Additional supplementary materials:  crystallographic information; 3D view; checkCIF report

## Figures and Tables

**Fig. 1.**
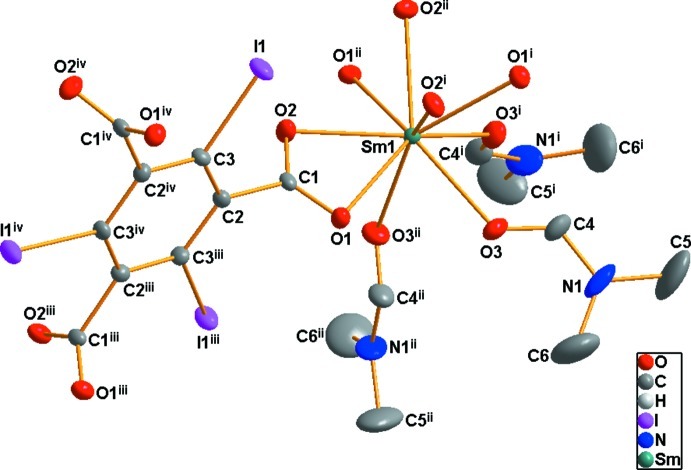
A portion of the crystal structure of (I) showing the coordination environment of Sm1, 30% probability displacement ellipsoids and atomic numbering [symmetry codes: (i) 1 - x, 1/2 + y, 3/2 - z; (ii) 3/2 - x, 1 - y, -1/2 + z; (iii) 3/2 - x, 1 - y, -1/2 + z; (iv) 1/2 - x, -y, 1/2 + z].

## supplementary crystallographic information

### Comment

Metal-organic framework (MOF) compounds have attracted considerable interest because of their potential applications in a variety of areas, including catalysis, shape-selective adsorption, gas storage, photochemistry, and materials with magnetic properties (Nakanishi *et al.*, 2007; Phan *et al.*, 2010; Suib *et al.*, 2008). The design and synthesis of MOFs with great potential for chemical and structural diversity, is one of the major current challenges in inorganic chemistry. To the best of our knowledge, MOF structure of the I_3_BTC ligand is not reported so far. The I_3_BTC ligand, besides three iodine atoms at the 2,4,6-sites of benzene ring, is same as 1,3,5- benzenetricarboxylic acid (H_3_BTC). Although the coordinated ability of carbonylic O atoms is influenced by the electronic properties of iodine atoms to some extent, its coordinated mode is almost no change (Daiguebonne *et al.*, 2002). In recent years, the construction of MOFs based on H_3_BTC ligand has been widely investigated owing to their fascinating coordination modes (Han *et al.*, 2012; Lu *et al.*, 2008; Serre *et al.*, 2004). Herein we report the hydrothermal synthesis and crystal structure of the title compound (I).

In (I), the asymmetric unit contains one Sm^III^ ion and one I_3_BTC anion, both situated on a threefold axis, and one DMF molecule in general position. As shown in Fig. 1, each Sm center is coordinated by nine O atoms -six carboxylate O atoms from three ligands and three O atoms from three DMF molecules. The Sm1–O bond lengths fall in the range of 2.446 (3)–2.562 (3) Å, and the O–Sm–O angles varying from 51.702 (88)–142.712 (91)°, thus falling in the expected region. Each ligand I_3_BTC bridges three Sm atoms to produce a three-dimensional metal organic framework structure, while coordinated solvent molecules (DMF) exist among the pore canals by coordinating O atoms to central metal ions.

### Experimental

The title compound was prepared by the solvothermal reaction of Sm(NO_3_)_3_.6H_2_O (100 mg), 2,4,6-triiodobenzene-1,3,5-tricarboxylic acid (100 mg), DMF (4 ml) and ethanol (4 ml) at 90 °C for 72 h. The autoclave was subsequently allowed to cool to room temperature. After washing with ethanol, colourless block crystals were obtained.

### Refinement

All H atoms were placed in geometrically calculated positions (C—H = 0.93–0.96 %A), and refined using a riding model, with U_iso_(H) = 1.2–1.5 U_eq_(C).

### Crystal data

**Table:**

[Sm(C_9_I_3_O_6_)(C_3_H_7_NO)_3_]	*D*_x_ = 2.245 Mg m^−^^3^
*M**_r_* = 954.43	Mo *K*α radiation, λ = 0.71073 Å
Cubic, *P*2_1_3	Cell parameters from 2685 reflections
Hall symbol: P 2ac 2ab 3	θ = 3.2–27.2°
*a* = 14.1341 (16) Å	µ = 5.41 mm^−^^1^
*V* = 2823.6 (6) Å^3^	*T* = 295 K
*Z* = 4	Prism, yellow
*F*(000) = 1772	0.16 × 0.15 × 0.15 mm

### Data collection

**Table:**

Bruker APEXII CCD area-detector diffractometer	2143 independent reflections
Radiation source: fine-focus sealed tube	2026 reflections with *I* > 2σ(*I*)
Graphite monochromator	*R*_int_ = 0.020
φ and ω scans	θ_max_ = 27.4°, θ_min_ = 3.2°
Absorption correction: multi-scan (*SADABS*; Bruker, 2005)	*h* = −12→7
*T*_min_ = 0.478, *T*_max_ = 0.498	*k* = 0→18
5119 measured reflections	*l* = −16→12

### Refinement

**Table:**

Refinement on *F*^2^	Hydrogen site location: inferred from neighbouring sites
Least-squares matrix: full	H-atom parameters constrained
*R*[*F*^2^ > 2σ(*F*^2^)] = 0.021	*w* = 1/[σ^2^(*F*_o_^2^) + (0.0236*P*)^2^] where *P* = (*F*_o_^2^ + 2*F*_c_^2^)/3
*wR*(*F*^2^) = 0.049	(Δ/σ)_max_ = 0.002
*S* = 1.03	Δρ_max_ = 0.72 e Å^−^^3^
2143 reflections	Δρ_min_ = −0.62 e Å^−^^3^
106 parameters	Extinction correction: *SHELXL97* (Sheldrick, 2008), Fc^*^=kFc[1+0.001xFc^2^λ^3^/sin(2θ)]^-1/4^
0 restraints	Extinction coefficient: 0.00049 (7)
Primary atom site location: structure-invariant direct methods	Absolute structure: Flack (1983), 943 Friedel pairs
Secondary atom site location: difference Fourier map	Flack parameter: 0.02 (2)

### Special details

**Table:**

Geometry. All e.s.d.'s (except the e.s.d. in the dihedral angle between two l.s. planes) are estimated using the full covariance matrix. The cell e.s.d.'s are taken into account individually in the estimation of e.s.d.'s in distances, angles and torsion angles; correlations between e.s.d.'s in cell parameters are only used when they are defined by crystal symmetry. An approximate (isotropic) treatment of cell e.s.d.'s is used for estimating e.s.d.'s involving l.s. planes.
Refinement. Refinement of *F*^2^ against ALL reflections. The weighted *R*-factor *wR* and goodness of fit *S* are based on *F*^2^, conventional *R*-factors *R* are based on *F*, with *F* set to zero for negative *F*^2^. The threshold expression of *F*^2^ > σ(*F*^2^) is used only for calculating *R*-factors(gt) *etc*. and is not relevant to the choice of reflections for refinement. *R*-factors based on *F*^2^ are statistically about twice as large as those based on *F*, and *R*- factors based on ALL data will be even larger. *SHELXTL* (Sheldrick, 2008)

### Fractional atomic coordinates and isotropic or equivalent isotropic displacement parameters (Å^2^)

**Table:**

	*x*	*y*	*z*	*U*_iso_*/*U*_eq_	
O2	0.9837 (2)	−0.17185 (19)	0.4744 (2)	0.0341 (7)	
C1	0.9099 (3)	−0.1640 (3)	0.4268 (3)	0.0219 (8)	
C2	0.8710 (2)	−0.2536 (3)	0.3796 (3)	0.0227 (8)	
C3	0.9151 (3)	−0.2914 (3)	0.2996 (3)	0.0236 (8)	
C4	0.8963 (4)	0.2099 (3)	0.4875 (4)	0.0464 (12)	
H4	0.9575	0.2290	0.5023	0.056*	
C5	0.8521 (6)	0.3734 (5)	0.5058 (7)	0.125 (4)	
H5A	0.8541	0.4090	0.4479	0.187*	
H5B	0.9125	0.3772	0.5367	0.187*	
H5C	0.8042	0.3990	0.5465	0.187*	
C6	0.7318 (5)	0.2501 (7)	0.4593 (7)	0.126 (4)	
H6A	0.7297	0.1860	0.4370	0.189*	
H6B	0.7099	0.2918	0.4103	0.189*	
H6C	0.6920	0.2564	0.5139	0.189*	
I1	1.03806 (2)	−0.22509 (2)	0.248693 (19)	0.03739 (10)	
N1	0.8303 (4)	0.2748 (3)	0.4848 (4)	0.0687 (14)	
O1	0.8674 (2)	−0.08834 (19)	0.4139 (2)	0.0304 (6)	
O3	0.8828 (2)	0.1250 (2)	0.4717 (2)	0.0428 (8)	
Sm1	0.999507 (13)	0.000493 (13)	0.500493 (13)	0.01931 (8)	

### Atomic displacement parameters (Å^2^)

**Table:**

	*U*^11^	*U*^22^	*U*^33^	*U*^12^	*U*^13^	*U*^23^
O2	0.0346 (17)	0.0259 (14)	0.0419 (17)	0.0000 (12)	−0.0116 (13)	−0.0055 (12)
C1	0.0222 (19)	0.0212 (19)	0.0224 (19)	−0.0053 (15)	0.0077 (15)	−0.0030 (15)
C2	0.0207 (19)	0.022 (2)	0.025 (2)	−0.0016 (15)	−0.0020 (14)	−0.0003 (15)
C3	0.0211 (19)	0.026 (2)	0.0234 (19)	−0.0046 (15)	0.0047 (15)	0.0007 (15)
C4	0.050 (3)	0.042 (3)	0.047 (3)	0.022 (2)	0.000 (2)	0.005 (2)
C5	0.170 (9)	0.061 (5)	0.143 (8)	0.061 (5)	−0.040 (7)	−0.039 (5)
C6	0.072 (5)	0.147 (10)	0.160 (9)	0.056 (6)	−0.011 (5)	−0.011 (6)
I1	0.03269 (16)	0.04066 (18)	0.03881 (18)	−0.01484 (12)	0.01194 (11)	−0.00927 (13)
N1	0.086 (4)	0.053 (3)	0.067 (3)	0.046 (3)	0.003 (3)	−0.007 (2)
O1	0.0303 (16)	0.0243 (15)	0.0365 (17)	0.0005 (12)	−0.0038 (13)	−0.0024 (12)
O3	0.0369 (18)	0.0390 (19)	0.053 (2)	0.0154 (14)	0.0044 (15)	0.0053 (16)
Sm1	0.01931 (8)	0.01931 (8)	0.01931 (8)	0.00093 (7)	0.00093 (7)	−0.00093 (7)

### Geometric parameters (Å, º)

**Table:**

O2—C1	1.245 (5)	C5—H5C	0.9600
O2—Sm1	2.474 (3)	C6—N1	1.480 (10)
C1—O1	1.240 (5)	C6—H6A	0.9600
C1—C2	1.534 (5)	C6—H6B	0.9600
C1—Sm1	2.844 (4)	C6—H6C	0.9600
C2—C3^i^	1.393 (5)	O1—Sm1	2.562 (3)
C2—C3	1.397 (5)	O3—Sm1	2.446 (3)
C3—C2^ii^	1.393 (5)	Sm1—O3^iii^	2.446 (3)
C3—I1	2.101 (4)	Sm1—O3^iv^	2.446 (3)
C4—O3	1.236 (6)	Sm1—O2^iv^	2.474 (3)
C4—N1	1.309 (6)	Sm1—O2^iii^	2.474 (3)
C4—H4	0.9300	Sm1—O1^iii^	2.562 (3)
C5—N1	1.458 (9)	Sm1—O1^iv^	2.562 (3)
C5—H5A	0.9600	Sm1—C1^iv^	2.844 (4)
C5—H5B	0.9600	Sm1—C1^iii^	2.844 (4)
			
C1—O2—Sm1	93.9 (2)	O3^iv^—Sm1—O1	73.51 (10)
O1—C1—O2	124.2 (4)	O2^iv^—Sm1—O1	133.46 (10)
O1—C1—C2	118.3 (3)	O2—Sm1—O1	51.69 (9)
O2—C1—C2	117.5 (3)	O2^iii^—Sm1—O1	71.50 (9)
O1—C1—Sm1	64.2 (2)	O3^iii^—Sm1—O1^iii^	77.37 (10)
O2—C1—Sm1	60.20 (19)	O3—Sm1—O1^iii^	73.51 (10)
C2—C1—Sm1	173.6 (2)	O3^iv^—Sm1—O1^iii^	142.72 (11)
C3^i^—C2—C3	118.3 (4)	O2^iv^—Sm1—O1^iii^	71.50 (9)
C3^i^—C2—C1	121.2 (3)	O2—Sm1—O1^iii^	133.46 (10)
C3—C2—C1	120.5 (3)	O2^iii^—Sm1—O1^iii^	51.69 (9)
C2^ii^—C3—C2	121.7 (4)	O1—Sm1—O1^iii^	118.13 (3)
C2^ii^—C3—I1	119.9 (3)	O3^iii^—Sm1—O1^iv^	73.51 (10)
C2—C3—I1	118.4 (3)	O3—Sm1—O1^iv^	142.72 (11)
O3—C4—N1	124.4 (5)	O3^iv^—Sm1—O1^iv^	77.37 (10)
O3—C4—H4	117.8	O2^iv^—Sm1—O1^iv^	51.69 (9)
N1—C4—H4	117.8	O2—Sm1—O1^iv^	71.50 (9)
N1—C5—H5A	109.5	O2^iii^—Sm1—O1^iv^	133.46 (10)
N1—C5—H5B	109.5	O1—Sm1—O1^iv^	118.13 (3)
H5A—C5—H5B	109.5	O1^iii^—Sm1—O1^iv^	118.13 (3)
N1—C5—H5C	109.5	O3^iii^—Sm1—C1	152.70 (11)
H5A—C5—H5C	109.5	O3—Sm1—C1	103.10 (11)
H5B—C5—H5C	109.5	O3^iv^—Sm1—C1	77.91 (10)
N1—C6—H6A	109.5	O2^iv^—Sm1—C1	110.46 (10)
N1—C6—H6B	109.5	O2—Sm1—C1	25.90 (10)
H6A—C6—H6B	109.5	O2^iii^—Sm1—C1	77.28 (10)
N1—C6—H6C	109.5	O1—Sm1—C1	25.84 (10)
H6A—C6—H6C	109.5	O1^iii^—Sm1—C1	128.98 (10)
H6B—C6—H6C	109.5	O1^iv^—Sm1—C1	95.43 (10)
C4—N1—C5	120.9 (6)	O3^iii^—Sm1—C1^iv^	77.91 (10)
C4—N1—C6	120.8 (6)	O3—Sm1—C1^iv^	152.70 (11)
C5—N1—C6	118.3 (5)	O3^iv^—Sm1—C1^iv^	103.10 (11)
C1—O1—Sm1	89.9 (2)	O2^iv^—Sm1—C1^iv^	25.90 (10)
C4—O3—Sm1	124.4 (3)	O2—Sm1—C1^iv^	77.28 (10)
O3^iii^—Sm1—O3	75.35 (12)	O2^iii^—Sm1—C1^iv^	110.46 (10)
O3^iii^—Sm1—O3^iv^	75.35 (12)	O1—Sm1—C1^iv^	128.98 (10)
O3—Sm1—O3^iv^	75.35 (12)	O1^iii^—Sm1—C1^iv^	95.43 (10)
O3^iii^—Sm1—O2^iv^	82.62 (10)	O1^iv^—Sm1—C1^iv^	25.84 (10)
O3—Sm1—O2^iv^	141.85 (10)	C1—Sm1—C1^iv^	103.16 (9)
O3^iv^—Sm1—O2^iv^	128.50 (10)	O3^iii^—Sm1—C1^iii^	103.10 (11)
O3^iii^—Sm1—O2	141.85 (10)	O3—Sm1—C1^iii^	77.91 (10)
O3—Sm1—O2	128.50 (10)	O3^iv^—Sm1—C1^iii^	152.70 (11)
O3^iv^—Sm1—O2	82.62 (10)	O2^iv^—Sm1—C1^iii^	77.28 (10)
O2^iv^—Sm1—O2	87.46 (10)	O2—Sm1—C1^iii^	110.46 (10)
O3^iii^—Sm1—O2^iii^	128.50 (10)	O2^iii^—Sm1—C1^iii^	25.90 (10)
O3—Sm1—O2^iii^	82.62 (10)	O1—Sm1—C1^iii^	95.43 (10)
O3^iv^—Sm1—O2^iii^	141.85 (10)	O1^iii^—Sm1—C1^iii^	25.84 (10)
O2^iv^—Sm1—O2^iii^	87.46 (10)	O1^iv^—Sm1—C1^iii^	128.98 (10)
O2—Sm1—O2^iii^	87.46 (10)	C1—Sm1—C1^iii^	103.16 (9)
O3^iii^—Sm1—O1	142.72 (11)	C1^iv^—Sm1—C1^iii^	103.16 (9)
O3—Sm1—O1	77.37 (10)		
			
Sm1—O2—C1—O1	−5.3 (4)	C1—O1—Sm1—O3^iv^	−96.3 (2)
Sm1—O2—C1—C2	173.3 (3)	C1—O1—Sm1—O2^iv^	31.5 (3)
O1—C1—C2—C3^i^	−76.0 (5)	C1—O1—Sm1—O2	−2.7 (2)
O2—C1—C2—C3^i^	105.3 (4)	C1—O1—Sm1—O2^iii^	99.1 (2)
Sm1—C1—C2—C3^i^	172 (2)	C1—O1—Sm1—O1^iii^	122.17 (19)
O1—C1—C2—C3	104.2 (4)	C1—O1—Sm1—O1^iv^	−31.0 (3)
O2—C1—C2—C3	−74.4 (5)	C1—O1—Sm1—C1^iv^	−2.6 (3)
Sm1—C1—C2—C3	−8 (3)	C1—O1—Sm1—C1^iii^	109.2 (3)
C3^i^—C2—C3—C2^ii^	2.2 (7)	O1—C1—Sm1—O3^iii^	88.8 (3)
C1—C2—C3—C2^ii^	−178.0 (3)	O2—C1—Sm1—O3^iii^	−86.3 (3)
C3^i^—C2—C3—I1	−177.81 (18)	C2—C1—Sm1—O3^iii^	−156 (2)
C1—C2—C3—I1	2.0 (5)	O1—C1—Sm1—O3	5.5 (3)
O3—C4—N1—C5	179.1 (7)	O2—C1—Sm1—O3	−169.7 (2)
O3—C4—N1—C6	−1.6 (9)	C2—C1—Sm1—O3	120 (2)
O2—C1—O1—Sm1	5.1 (4)	O1—C1—Sm1—O3^iv^	77.1 (2)
C2—C1—O1—Sm1	−173.4 (3)	O2—C1—Sm1—O3^iv^	−98.1 (2)
N1—C4—O3—Sm1	−170.0 (4)	C2—C1—Sm1—O3^iv^	−168 (2)
C4—O3—Sm1—O3^iii^	33.2 (4)	O1—C1—Sm1—O2^iv^	−156.1 (2)
C4—O3—Sm1—O3^iv^	111.6 (4)	O2—C1—Sm1—O2^iv^	28.8 (2)
C4—O3—Sm1—O2^iv^	−23.6 (5)	C2—C1—Sm1—O2^iv^	−41 (2)
C4—O3—Sm1—O2	179.4 (4)	O1—C1—Sm1—O2	175.1 (4)
C4—O3—Sm1—O2^iii^	−99.8 (4)	C2—C1—Sm1—O2	−70 (2)
C4—O3—Sm1—O1	−172.4 (4)	O1—C1—Sm1—O2^iii^	−73.7 (2)
C4—O3—Sm1—O1^iii^	−47.7 (4)	O2—C1—Sm1—O2^iii^	111.1 (3)
C4—O3—Sm1—O1^iv^	67.3 (4)	C2—C1—Sm1—O2^iii^	41 (2)
C4—O3—Sm1—C1	−174.9 (4)	O2—C1—Sm1—O1	−175.1 (4)
C4—O3—Sm1—C1^iv^	21.3 (5)	C2—C1—Sm1—O1	115 (2)
C4—O3—Sm1—C1^iii^	−74.0 (4)	O1—C1—Sm1—O1^iii^	−73.78 (19)
C1—O2—Sm1—O3^iii^	132.2 (2)	O2—C1—Sm1—O1^iii^	111.1 (2)
C1—O2—Sm1—O3	12.9 (3)	C2—C1—Sm1—O1^iii^	41 (2)
C1—O2—Sm1—O3^iv^	77.5 (2)	O1—C1—Sm1—O1^iv^	152.9 (2)
C1—O2—Sm1—O2^iv^	−153.2 (2)	O2—C1—Sm1—O1^iv^	−22.3 (2)
C1—O2—Sm1—O2^iii^	−65.6 (3)	C2—C1—Sm1—O1^iv^	−92 (2)
C1—O2—Sm1—O1	2.7 (2)	O1—C1—Sm1—C1^iv^	177.9 (2)
C1—O2—Sm1—O1^iii^	−91.8 (3)	O2—C1—Sm1—C1^iv^	2.8 (3)
C1—O2—Sm1—O1^iv^	156.6 (2)	C2—C1—Sm1—C1^iv^	−67 (2)
C1—O2—Sm1—C1^iv^	−177.2 (3)	O1—C1—Sm1—C1^iii^	−75.0 (3)
C1—O2—Sm1—C1^iii^	−77.76 (18)	O2—C1—Sm1—C1^iii^	109.90 (18)
C1—O1—Sm1—O3^iii^	−130.8 (2)	C2—C1—Sm1—C1^iii^	40 (2)
C1—O1—Sm1—O3	−174.6 (3)		

Symmetry codes: (i) −*y*+1/2, −*z*, *x*−1/2; (ii) *z*+1/2, −*x*+1/2, −*y*; (iii) −*z*+3/2, −*x*+1, *y*+1/2; (iv) −*y*+1, *z*−1/2, −*x*+3/2.
